# Diagnostic accuracy of low-radiation coronary computed tomography angiography with low tube voltage and knowledge-based model reconstruction

**DOI:** 10.1038/s41598-018-37870-3

**Published:** 2019-02-04

**Authors:** Joohee Lee, Tae Hoon Kim, Byoung Kwon Lee, Young Won Yoon, Hyuck Moon Kwon, Bum Kee Hong, Pil-Ki Min, Eui-Young Choi, Chi Suk Oh, Chul Hwan Park

**Affiliations:** 1 Department of Radiology and Research Institute of Radiological Science, Gangnam Severance Hospital, Yonsei University College of Medicine, Seoul, Republic of Korea; 2 Division of Cardiology, Heart Center, Gangnam Severance Hospital, Yonsei University College of Medicine, Seoul, Republic of Korea

## Abstract

We aimed to evaluate the accuracy of coronary computed tomography angiography (CCTA) with a low-radiation protocol and iterative model reconstruction (IMR), in comparison with invasive coronary angiography (ICA). Sixty-one patients (45 males; mean age, 61.9 ± 9.2 years) with suspected coronary artery disease who underwent CCTA and ICA were retrospectively enrolled. CCTA was performed with low tube voltage (80 or 100 kVp), low tube current (100–200 mAs), prospective ECG triggering, and IMR using a 64-slice computed tomography scanner. Coronary artery disease was defined as luminal narrowing of >50%, as assessed using CCTA and ICA. The sensitivity, specificity, positive (PPV) and negative (NPV) predictive value, and accuracy of CCTA were examined. The mean radiation dose of CCTA was 1.05 ± 0.36 mSv. No non-diagnostic segment was noted. The sensitivity, specificity, PPV, NPV, and accuracy of CCTA were 86.4%, 96.1%, 80.3%, 97.5%, and 94.6% on a per segment basis, 93.1%, 94.7%, 88.3%, 97.0%, and 94.2% on a per vessel basis, and 100%, 83.3%, 93.5%, 100%, and 95.1% on a per patient basis, respectively. In conclusion, a low-radiation CCTA protocol with IMR may be useful for diagnosing coronary artery disease, as it reduces the radiation dose while maintaining diagnostic accuracy.

## Introduction

Coronary computed tomographic angiography (CCTA) is a useful, non-invasive diagnostic imaging modality for the detection of coronary artery disease (CAD). Various strategies and scanning protocols enable a reduction in the radiation dose used for CCTA, including high-pitch spiral acquisition, prospective ECG triggering, tube voltage lowering, and tube current modulation^1–5^. The diagnostic performance of prospectively ECG-triggered CCTA with lowered radiation exposure by decreasing the scanning duration showed a high negative predictive value (NPV), when using stenosis by invasive coronary angiography (ICA) as the reference in patients with low-to-intermediate probability for CAD^6^.

Recently, low-radiation CCTA has been used to reduce radiation exposure with the low tube voltage method. However, increased noise is a problem of this technique, which can affect image quality and diagnostic performance. The newly introduced, knowledge-based iterative model reconstruction (IMR) with a fully iterative algorithm can now be clinically used to significantly reduce noise, while maintaining image quality, as compared to iterative reconstruction (IR) with tube voltage lowering^7,8^. To our knowledge, a few recent studies have evaluated the diagnostic performance and clinical feasibility of CCTA for CAD using low tube voltage and the model-based iterative reconstruction algorithm (MBIR), which is not yet commercially available because of the extended reconstruction time; these studies have showed relatively low specificity^9,10^. In contrast, IMR can be used in daily practice; however, while the good image quality of low-dose CCTA with IMR has been reported^7,8^, its diagnostic accuracy has not been well-established.

Here, we aimed to assess the accuracy of CCTA using low tube voltage, the IMR technique, prospective ECG triggering, and a radiation dose of approximately 1 mSv, in comparison with ICA, in patients with suspected CAD. In particular, we evaluated the diagnostic accuracy on a per-segment, per-vessel, and per-patient basis, and determined its impact and the factors underlying misdiagnosis on CT images.

## Results

### Characteristics of the subjects and coronary computed tomography angiography

In this study, we examined a total of 61 subjects (male:female, 45:16; mean age, 61.9 ± 9.2 years). All CCTA procedures were performed without complications. The mean radiation dose of the 61 patients was 1.05 ± 0.36 mSv (Table 1). The mean CT attenuation value of the aortic root on CCTA was 469.1 ± 67.8 Hounsfield units (HU). The mean image noise was 31.8 ± 8.6. The signal-to-noise ratio (SNR) at the right coronary artery (RCA), left main coronary artery (LM), left anterior descending artery (LAD) and left circumflex artery (LCx) were 15.38, 14.37, 13.86 and 14.27, respectively. The contrast-to-noise ratio (CNR) at the RCA, LM, LAD and LCx were 18.25, 17.28, 16.65 and 17.02, respectively.

**Table 1:** Coronary computed tomographic angiography parameters and radiation dosage.

Tube voltage	Subject number	Radiation dose (mSv)
Total	61	1.05 ± 0.36
100 kVp	33	1.36 ± 0.14
80 kVp	28	0.71 ± 0.15

Data are presented as mean ± standard deviation.

### Qualitative analysis

A total of 768 segments with diameters >1.5 mm were evaluated. A total of 391 segments (50.9%) were considered excellent (Grade 4), 293 segments (38.2%) were considered good (Grade 3), and 84 segments (10.9%) were considered adequate (Grade 2). None of the segments were considered non-diagnostic (Grade 1). The mean image quality was 3.40 ± 0.68. On per-segment analysis, the image quality of the RCA, LM, LAD, and LCx were found to be 3.70 ± 0.53, 3.90 ± 0.35, 3.41 ± 0.62, and 3.35 ± 0.71, respectively (Table 2). The inter-reader agreement for the image quality assessment was 0.791 for RCA, 0.835 for LM, 0.751 for LAD and 0.709 for LCx.

**Table 2:** Quantitative and qualitative analysis of the CCTA images.

	61 Patients
Attenuation of the aortic root	469.1 ± 67.8 HU
Noise	31.8 ± 8.6
**SNR**	
RCA^a^	15.38 ± 2.80
LM^b^	14.37 ± 2.53
LAD^c^	13.86 ± 2.56
LCx^d^	14.27 ± 3.02
**CNR**	
RCA	18.25 ± 2.76
LM	17.28 ± 2.64
LAD	16.65 ± 2.55
LCx	17.02 ± 3.09
**Image quality**	
Per-vessel analysis	3.40 ± 0.68
RCA	3.70 ± 0.53
LM	3.90 ± 0.35
LAD	3.41 ± 0.62
LCx	3.35 ± 0.71
**No. of segments**	
No. of segments in each score (%)	768 (100)
Grade 4 (Excellent)	391 (50.9)
Grade 3 (Good)	293 (38.2)
Grade 2 (Adequate)	84 (10.9)
Grade 1 (Poor)	0 (0)

Data are presented as mean ± standard deviation. Abbreviations: ^a^Right coronary artery, ^b^Left main coronary artery, ^c^Left anterior descending artery, ^d^Left circumflex artery. Segments with a diameter of at least 1.5 mm at the origin were included.

CCTA: coronary computed tomographic angiography.

### Per-segment analysis

**Figure 1:**
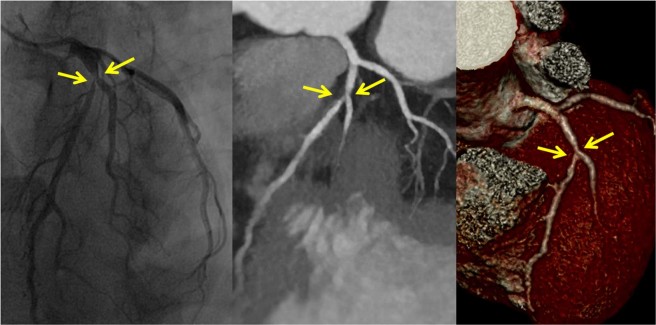
True positive finding with left anterior descending stenosis. Proximal left anterior descending stenosis in a 53-year-old woman (effective radiation dose, 0.53 mSv; BMI, 20.3 kg/m^2^; mean heart rate, 60 bpm), diagnosed via invasive coronary angiography (**a**), and examined via coronary computed tomographic angiography under 80 kVp/100 mAs, with maximum intensity projection (**b**) and volume-rendered (**c**) images and iterative model reconstruction algorithm reconstruction.

Of the 768 segments, 118 showed significant stenosis on ICA, including 102 with true positive results (Fig. 1) and 16 with false negative results on CCTA. Of the 650 segments that did not show significant stenosis on ICA, 625 exhibited true negative results on CCTA. The sensitivity, specificity, positive predictive value (PPV), NPV, and overall accuracy of CCTA were 86.4%, 96.1%, 80.3%, 97.5%, and 94.6%, respectively (Table 3).

**Table 3:** Diagnostic accuracy of CCTA.

	Sensitivity	Specificity	PPV	NPV	Accuracy
Per segment analysis	86.4%	96.1%	80.3%	97.5%	94.6%
Per vessel analysis	93.1%	94.7%	88.3%	97.0%	94.2%
Per patients analysis	100%	83.3%	93.5%	100%	95.1%

### Per-vessel analysis

Of the 244 vessels, 73 showed significant stenosis on ICA, including 68 with true positive results and 5 with false negative results on CCTA. Of the 162 vessels that did not show significant stenosis on ICA, 162 exhibited true negative results on CCTA. The sensitivity, specificity, PPV, NPV, and overall accuracy of CCTA were 93.1%, 94.7%, 88.3%, 97.0%, and 94.2%, respectively (Table 3).

### Per-patient analysis

Of the 61 patients, 43 showed significant stenosis on ICA, including 43 with true positive results and none with false negative results on CCTA. Of the 18 patients who did not show significant stenosis on ICA, 15 exhibited true negative results on CCTA. The sensitivity, specificity, PPV, NPV, and overall accuracy of CCTA were 100%, 83.3%, 93.5%, 100%, and 95.1%, respectively (Table 3).

### Analysis of false positive segments and false negative segments

**Figure 2:**
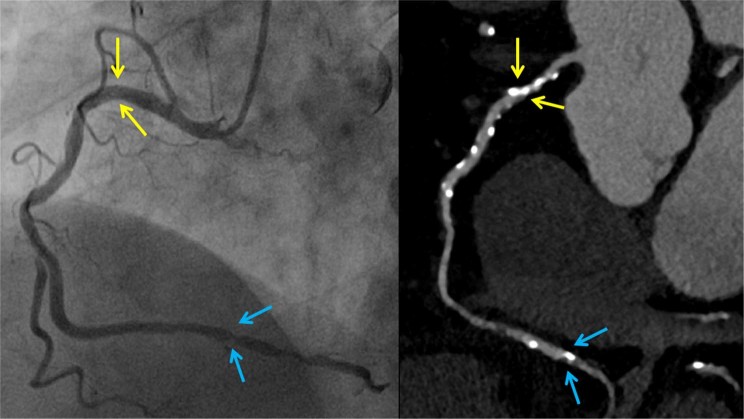
False positive finding. Overestimated (false positive) proximal right coronary artery (RCA) stenosis on coronary computed tomographic angiography (CCTA) under 100 kVp/150 mAs in a 66-year-old man (effective radiation dose, 1.64 mSv; BMI, 26.1 kg/m^2^; mean heart rate, 63 bpm). Invasive coronary angiography (ICA) of the RCA confirmed the insignificant stenosis (**a**). Curved multiplanar reformation of the RCA shows significant stenosis in the proximal segment caused by a calcified plaque (**b**), although this was a false positive finding as compared to the ICA. In the same vessel, distal right coronary stenosis was concordantly detected (true positive) via CCTA and ICA.

**Figure 3:**
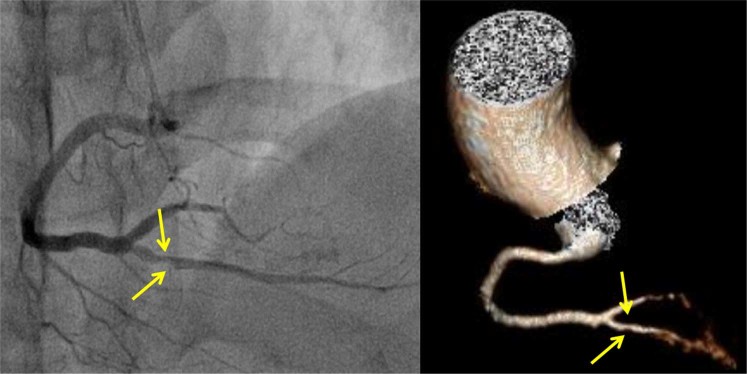
False negative finding. Right posterior descending artery stenosis, detected via invasive coronary angiography (**a**), and underestimated (false negative) via coronary computed tomographic angiography (**b**) under 100 kVp/150 mAs in a 66-year-old woman (effective radiation dose, 1.32 mSv; BMI, 24.0 kg/m^2^, mean heart rate, 58 bpm).

A total of 25 false positive segments (Fig. 2) were reported in 20 patients (see Supplementary Table S1). These findings were primarily affected by the blooming artifact as a result of dense calcification, the streak artifact that reduced the effective attenuation adjacent to the dense calcification, and the partial volume effect with the adjacent vessel. The 16 false negative cases (Fig. 3) in 13 patients were primarily a result of poor vessel attenuation at the distal small branches and eccentrically calcified plaques in the vessel wall (see Supplementary Table S2).

## Discussion

In this study, we sought to assess the utility of prospective CCTA with low tube voltage and IMR reconstruction for the detection of CAD. Compared with the reference standard ICA, the present study showed that CCTA had high diagnostic performance in ruling out CAD, along with a marked dose reduction at a 1 mSv fraction of effective radiation.

CCTA has become increasingly used as a useful, non-invasive diagnostic imaging modality for detecting CAD. The very high sensitivities and specificities of prospectively ECG-triggered CCTA, which range from 98% to 100% and 86% to 94%, respectively, support its use as a reliable modality for CAD diagnosis^6,11^. Moreover, the very high NPV suggests that CCTA can be most suitably used for excluding CAD, particularly in populations with low-to-intermediate risk^12^.

However, the issue concerning the radiation dose during CCTA is important, considering the potential stochastic cancer risks associated with medical radiation exposure^13^. Compared to that of conventional retrospective ECG-gated scanning, the reported mean effective radiation dose of CCTA in prospectively ECG-triggered studies was significantly reduced by >60% (mean dose, 13.8 mSv vs. 4.5 mSv, respectively), whereas a reduction in the effective dose by 46% was observed in studies with scans performed at 100 kVp, as compared to scans performed at 120 kVp (mean dose, 1.65 mSv vs. 3.05 mSv, respectively)^11,14^. Furthermore, in a previous study, we found that the use of 80 kVp yielded a reduction in the mean radiation dose by nearly 50% to sub-mSv levels, as compared to CCTA at 100 kVp^15^.

IMR relies on statistical and system models, and views reconstruction as an optimization process. Newer techniques, such as the current one, may simultaneously provide further reduction in image noise, potential dose savings, and improved image quality under conditions of reduced tube voltage^16,17^. The relevant use of the lower kVp for CCTA as well as IMR can further provide reduced radiation doses without compromising image quality.

Our study with prospectively ECG-triggered CCTA at low kVp with the IMR technique showed clinically acceptable diagnostic accuracy, consistent with that in previous reports using other various reconstructive technologies for reducing radiation dose exposure. Based on our previous study, low-radiation CCTA using IMR is clinically feasible with appreciable image interpretability, and has the advantage of the executive time of reconstruction, as compared to other reports of the feasibility of submillisievert CCTA scanning with the latest-generation iterative reconstruction algorithms in clinical settings^9,10^.

In the present study, low-radiation CCTA (1.05 ± 0.36 mSv) with low tube voltage (80 or 100 kVp) and IMR yielded high sensitivity (86.4%), specificity (96.1%), PPV (80.3%), and NPV (97.5%) in a per segment-based analysis. These results are comparable to previous findings for CCTA obtained from prospectively triggered studies using the IR algorithm with showing high diagnostic accuracy (94.2% per vessel analysis) in patients with suspected CAD^6,11^. Previously reported CCTA with 80–100 kVp/150–210 mA scan protocol using MBIR showed 85% accuracy per vessel analysis^9^. Despite the reduced radiation dose (approximately 0.29 mSv), its extended reconstruction time causes difficulty in applying MBIR-CCTA in practice^9^.

Our findings suggest that the diagnostic accuracy of CCTA for the detection of coronary artery stenosis was affected by various factors, similar to the previously reported factors for misdiagnosis on CCTA. The false positive results were primarily affected by the blooming artifact due to a greater extent of dense calcification in coronary arteries (15 of 25 false positive segments, 60%). The presence of densely calcified plaques accentuated the partial volume averaging of different densities within a single voxel, thus leading to worse coronary lumen visualization due to insufficient spatial resolution and consequently to overestimation of luminal stenosis^18–20^. Although low tube voltage settings can increase the blooming artifacts from calcification, under low radiation dose conditions, the use of IMR algorithms for suppressing blooming artifacts in the presence of coronary calcium can reduce the calcium scores and image noise, in comparison with the traditional filtered back projection (FBP)^21,22^. However, this factor may still represent the major cause of false positive CCTA results and image artifacts. Another factor affecting false positive results, which are misinterpreted as non-calcified plaques, is the streak artifact as a result of X-ray beam penetrability, which reduces the effective attenuation adjacent to the dense calcification. In the present study, false negativities were primarily affected by inadequate vessel attenuation at the distal small branches, as a result of the limited spatial resolution on MDCT that causes the blurring of vessel margins (12 of 16 false-negative segments, 75%). The other 4 cases of false negative diagnoses were caused by eccentric calcifications on opposite sides of the vessel wall, which led to the underestimation of coronary artery stenosis severity, as reported in a previous study^18,23^.

Based on the analysis of CCTA results from patients who underwent ICA, low-dose CCTA may be suitable for excluding CAD in low-to-intermediate probability populations from the favorable NPV on per-patient, per-vessel, and per-segment bases. Moreover, there was an inverse correlation between sensitivity and specificity in comparison of segment-based and patient-based analysis (86.4%/96.1% vs. 100%/83.3%), consistent with the findings of other meta-analyses^24^. This finding may be related to the fact that one diseased segment is sufficient for detection of CAD in a patient, and that this patient may have additional diseased segments undetected on CCTA. Thus, CCTA may be currently suitable for excluding CAD at the patient level, or for detection of stenoses at the segment level^24,25^.

With these low-radiation CCTA protocols, it is also possible to reduce the amount of contrast agent administered, because the iodine attenuation increases with a decrease in the tube voltage as a result of contrast enhancement by reducing the energy of X-ray photons, and the mean k-absorption edge of iodine is closer to the reduced voltage^26,27^. Several reports have suggested that the contrast media dose at 80 kVp CCTA could be decreased via iodine enhancement, with acceptable image quality^26,28–30^. Low contrast-dose protocols could protect renal function by using a diagnostically appropriate amount of contrast medium for low-radiation CCTA. Therefore, there is a need for further investigation regarding low-radiation and low contrast-dose CCTA protocols with IMR reconstruction.

### Limitations

This study has certain limitations. First, the main limitation is the small sample size, retrospective design and small body size of the study subjects (mean BMI 24.4 kg/m^2^) in this single-center study. Second, there might be a referral bias because patients for whom CCTA excludes a stenosis are rarely sent for follow-up angiography, which means that lesions could have been missed. However, in this retrospective observational study, we recruited patients who underwent ICA in accordance with the relevant clinical indications. Third, the effective radiation dose could be further reduced if the tube current of the patients was more strictly modulated. We used the CCTA scan protocol with 80 kVp/100–200 mAs or 100 kVp/100–150 mAs, according to the BMI of the patients. The use of the IMR techniques could reduce image noise and permit further reduction of the radiation dose, as data can be acquired using a lower tube current (<100 mAs)^9^. Although the use of lower tube current results in noisy images, IMR could reduce the image noise without reducing their diagnostic quality.

Recently, a new conversion factor for estimating effective doses during CCTA was presented, such that a cardiac-specific conversion factor of 0.026 mSv/(mGy × cm) is appropriate to estimate effective radiation doses; this reflects a new tissue-weighting factor^31^. However, one of the primary goals of this study was to measure the efficacy of low-radiation CCTA using 80 kVp or 100 kVp and IMR reconstruction, showing similar diagnostic accuracy in comparison with other studies using different reconstruction method with the previous conversion factor. We calculated the effective radiation dose for CCTA by multiplying the dose-length product (DLP) with a conversion coefficient of 0.014 mSv/(mGy × cm). There is a need for further investigation regarding use of the new cardiac-specific factors to ensure more accurate dosimetry; this can contribute to benefit-risk calculus in cardiac imaging strategies, and support optimization of radiation safety for patients.

## Conclusions

In conclusion, a low-radiation CCTA protocol with low tube voltage, low tube current, prospective ECG triggering, and IMR could be a useful strategy for reducing the radiation dose of CCTA, while maintaining the diagnostic accuracy. Our findings suggest that IMR enables the accurate non-invasive diagnosis of CAD with CCTA at a 1 mSv fraction of effective radiation.

## Methods

This study was approved by Gangnam Severance Hospital ethics committee/institutional review board (3-2016-0282) and the requirement for written informed consent was waived due to the retrospective nature of this study. All methods were performed in accordance with the relevant guidelines and regulations.

### Study population

**Figure 4:**
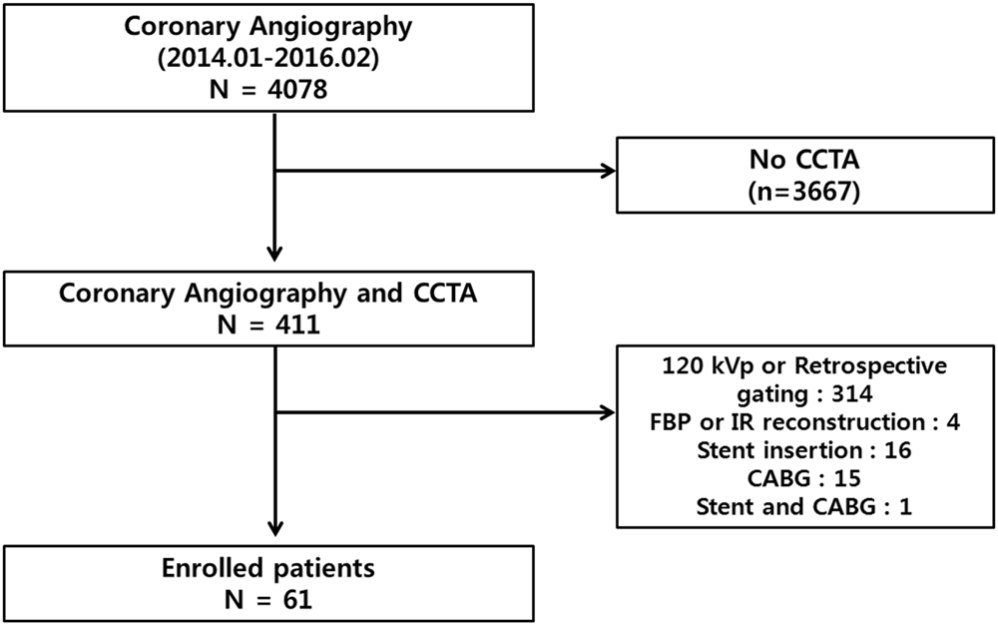
Flow chart of patient selection. Among the 4078 patients with suspected coronary artery disease (CAD) referred to undergo invasive coronary angiography (ICA), the cases of 411 patients who underwent coronary computed tomographic angiography (CCTA) within 2 months of ICA were reviewed. A total of 314 patients were excluded because they underwent CCTA using 120 kVp or because they underwent CT with retrospective ECG-gated data acquisition. Those who underwent CCTA using reconstructive methods such as traditional filtered back projection or hybrid iterative reconstruction (n = 4), or those who underwent CCTA for stent evaluation (n = 16), bypass graft evaluation (n = 15), or both (n = 1) were excluded. Finally, a total of 61 patients (45 men and 16 women; mean age, 61.9 ± 9.2 years) were enrolled.

The study was designed as a retrospective study. From January 2014 to February 2016, a total of 4078 patients with suspected CAD were referred to our hospital for ICA. Among these, 411 patients who underwent CCTA within 2 months prior to ICA were examined; 314 patients were excluded because they underwent CCTA at 120 kVp or because they underwent CT with retrospective ECG-gated data acquisition. Moreover, we excluded patients who underwent CCTA using reconstructive methods such as traditional FBP or hybrid IR (n = 4), or those who underwent CCTA for stent evaluation (n = 16), bypass graft evaluation (n = 15), or both (n = 1). Finally, a total of 61 patients (45 men and 16 women; mean age, 61.9 ± 9.2 years) were enrolled in our analysis (Fig. 4). Their clinical presenting symptoms included dyspnea, atypical chest pain, abnormal ECG or exercise test results, or atypical or typical angina pectoris. They all showed low-to-intermediate pre-test probability of CAD. The demographic data and cardiac risk factors for the patients are summarized in Table 4.

**Table 4:** Demographic data and baseline characteristics of 61 patients with coronary computed tomographic angiography (CCTA).

Variable	Value
Age, yrs	61.9 ± 9.2
Male/Female, n (%)	45 (73.7)/16 (26.3)
Hypertension, n (%)	39 (63.9)
Diabetes, n (%)	7 (11.5)
Smoking history: current/ex-smoker/never, n (%)	5 (8.2)/21 (34.4)/37 (60.6)
Height, cm	167.4 ± 7.7
Weight, kg	68.6 ± 9.5
Heart rate (bpm)	58.3 ± 4.8
Body mass index, kg/m^2^	24.4 ± 2.6

Data are presented as mean ± standard deviation.

### Coronary computed tomographic angiography protocol

All CCTA examinations were performed using one CT scanner (Ingenuity Core 128, Philips Healthcare, Cleveland, OH, USA) with prospective ECG triggering and a step-and-shoot axial scanning technique. CT scanning was performed from the carina to the diaphragm in a craniocaudal direction at the end-inspiratory pause. When the pre-examination heart rate was higher than 65 beats per min, a β-blocker (40–80 mg propranolol hydrochloride; Pranol, Daewoong Pharmaceutical Co., Ltd., Seoul, Korea) was administered per os 1 hour before the examination, without additional intravenous injection.

The CT scanning parameters were as follows: tube voltage and tube current, 80 kVp and 100–200 mAs or 100 kVp and 100–150 mAs (based on the body mass index [BMI]; 100 kVp for BMI > 25 kg/m^2^ and 80 kVp for BMI < 25 kg/m^2^); detector collimation with dynamic z-focal spot imaging, 64 × 0.625 mm; gantry rotation time, 400 ms; table feed per rotation, 4 cm; and center of the imaging window, 70–80% of the R-R interval.

Iodinated contrast agent (Optiray 350; Tyco Healthcare, Kantata, Canada), was administered intravenously via an antecubital 18-gauge catheter at a rate of 0.50–0.75 mL/kg for 15 s, followed by the administration of 50 mL of normal saline using a power injector (Dual Shot; Nemoto Kyorindo Co., Ltd., Tokyo, Japan). Data acquisition was started 7 s after the trigger threshold of 130 HU was achieved at the proximal descending aorta using the real-time bolus tracking technique. All patients performed the breath-hold maneuver successfully. The effective radiation dose for CCTA was calculated by multiplying the dose-length product (DLP) with a conversion coefficient of 0.014 mSv/(mGy × cm).

### Computed tomography image reconstruction

CT images were reconstructed using iterative model reconstruction (IMR-level 1; Philips Healthcare). The images were sent to a picture archiving and communication system (PACS; Centricity 2.0, GE Medical Systems, Mount Prospect, IL, USA). The reconstruction parameters were as follows: slice thickness, 0.9 mm; increment, 0.45 mm; field of view, 15–23 cm; pixel image matrix, 512 × 512; and kernel, XCC. Post-processing and reconstruction were performed with multiplanar reformatted (MPR), curved planar reformatted (CPR), and maximum intensity projection (MIP) images using commercially available software (Aquarius Workstation V3.6, TeraRecon, San Mateo, CA, USA).

### Computed tomography data analysis

All images were reviewed and interpreted using the PACS workstation. Two radiologists (with more than 10 years of experience interpreting cardiac images) who were blinded to patient history and the results of coronary angiography evaluated the CCTA findings. Coronary arteries were divided into 18 segments according to the Society of Cardiovascular Computed Tomography guidelines^32^. The segments were included for analysis only if the diameter of the origin was equal or larger than 1.5 mm.

The vascular attenuation values were measured using a round region of interest larger than 1.5 cm^2^ at the ascending aorta. Image noise was evaluated in CCTA based on one standard deviation of the attenuation value at the ascending aorta. The SNR was calculated as vascular attenuation/image noise. The CNR was calculated as [(attenuation of vessel) - (attenuation of the adjacent perivascular fat)]/image noise.

The qualitative image quality of the 18 coronary segments was analyzed using a 4-point grading scale as follows: Grade 1 (poor/nondiagnostic), severe image degradation or discontinuation of vessel contour that prevented vessel lumen evaluation; Grade 2 (adequate), moderate image degradation that impeded vessel lumen evaluation; Grade 3 (good), minor image degradation that did not affect vessel lumen evaluation; and Grade 4 (excellent), no image degradation (Supplementary Figure)^23^.

All reconstructed images, including axial source images and MPR, CPR, and MIP images, were analyzed to evaluate stenosis. Half of the sum of the normal proximal and distal lumen within the same segment was used as the reference for percent stenosis. Luminal narrowing greater than 50% was regarded as significant stenosis. If there was more than one lesion within the same segment, the worst stenosis percentage was used for the analysis. The results of CCTA were compared to those obtained with ICA. Subsequently, two radiologists re-evaluated the cases for false positive and false negative segments, and the possible factors affecting diagnostic accuracy, such as location, vessel wall condition, coronary calcium scores, vessel attenuation, vessel wall motion, and other possible factors, in each corresponding segment, were discussed. Discrepancies, if any, were resolved by consensus.

### Invasive coronary angiography

ICA was performed with standard techniques via radial approach. All images were digitalized for offline analysis using commercially available software (CAAS II; Pie Medical Imaging, Maastricht, Netherlands). Segments were included for analysis at the bifurcation area only if the diameter was equal to or larger than 1.5 mm. Using at least 2 image planes, quantitative measurements were performed as follows: degree of stenosis (%) = [(the reference diameter - the minimal luminal diameter)/the reference diameter]*100. The reference diameter was determined as the mean of the normal proximal and distal lumen within the same segment. Each vessel was considered to have significant stenosis if the stenosis was greater than 50%.

### Statistical analysis

Continuous variables are expressed as mean ± standard deviation and categorical variables are expressed as frequencies or percentages. Shapiro-Wilk tests were performed to evaluate the distribution of the data Inter-reader agreement for the image quality assessment of CCTA was evaluated by using κ statistic. A κ value of 0.00–0.20 signified none to slight agreement; 0.21–0.40, fair agreement; 0.41–0.60, moderate agreement; 0.61–0.80, good agreement; and 0.81–1.00, excellent agreement. The sensitivity, specificity, PPV, NPV, and diagnostic accuracy of low-dose CCTA were calculated by using ICA as a reference, on a per-segment, per-vessel, and per-patient basis. All statistical analyses were performed with the SPSS 20 Statistical Package for the Social Sciences (Chicago, IL, U.S.A.).

## Supplementary information

Supplementary Tables and Figure
