# Supplementary material for: Diagnostic accuracy of low-radiation coronary computed tomography angiography with low tube voltage and knowledge-based model reconstruction
Source: Sci Rep. 2019 Feb 4;9:1308. doi: 10.1038/s41598-018-37870-3 (PMC6362232; doi:10.1038/s41598-018-37870-3)

Diagnostic accuracy of low-radiation coronary computed tomography angiography with low tube voltage and knowledge-based model reconstruction

Joohee Lee, M.D.[a], Tae Hoon Kim, M.D.[a], Byoung Kwon Lee, M.D.[b], Young Won Yoon, M.D.[b], Hyuck Moon Kwon, M.D.[b], Bum Kee Hong, M.D.[b], Pil-Ki Min, M.D.[b], Eui-Young Choi, M.D.[b], Chi Suk Oh[a], Chul Hwan Park, M.D.[a]

[a]Department of Radiology and Research Institute of Radiological Science, Gangnam Severance Hospital, Yonsei University College of Medicine, Republic of Korea

[b]Division of Cardiology, Heart Center, Gangnam Severance Hospital, Yonsei University College of Medicine, Seoul, Republic of Korea

Corresponding Author: Chul Hwan Park, M.D.

Address: Department of Radiology, Gangnam Severance Hospital

211 Eonjuro, Gangnam-Gu, Seoul 135-720, Republic of Korea

Tel: 82-2-2019-3510

Fax: 82-2-3462-5472

E-mail: park_chulhwan@yuhs.ac

Conflicts of Interest and Source of Funding: The authors declare no conflicts of interest. This research received no specific grant from any funding agency in the public, commercial, or not-for-profit sectors.

Short Title: Low voltage CCTA with model reconstruction

Supplementary Table S1. False positive findings and the factors affecting these CCTA results

Age(y)/ Sex	kVp	mAs	Aorta (HU)	Noise	Calcium score	Location	Affecting factor
**76/M**	100	150	431	28	254	LM[a]	Blooming artifact by dense calcified plaque
**67/M**	100	150	472	26	820	pLAD[b]	Blooming artifact by dense calcified plaque
**67/M**	80	200	535	24	923	mLAD[c]	Blooming artifact by dense calcified plaque
**61/M**	80	200	512	29	754	OM1[d]	Partial volume with adjacent vessel
**76/M**	100	150	505	31	365	dLCx[e]	Partial volume and motion artifact
**65/M**	80	200	656	24	1907	LM	Blooming artifact by dense calcified plaque
						pLAD	Blooming artifact by dense calcified plaque
						pLCx[f]	Blooming artifact by dense calcified plaque
**65/M**	100	150	266	30	1191	LM	Blooming artifact by dense calcified plaque
**59/F**	80	150	470	49	206	D1[g]	Partial volume with adjacent vessel
							Low attenuation
**78/M**	100	150	485	29	53	pLAD	Blooming artifact by dense calcified plaque
**51/M**	100	150	387	29	102	OM1	Blooming artifact by dense calcified plaque
**73/F**	100	150	463	38	192	pLAD	Blooming artifact by dense calcified plaque
**64/M**	80	150	418	41	0	mLAD	Partial volume with adjacent vessel
							Low attenuation
**58/M**	80	100	527	37	0	D1	Blooming artifact by dense calcified plaque
							Low attenuation

**70/F**	80	150	497	32	223	dLCx	Low attenuation
**60/M**	100	150	426	22	594	pLAD	Streak artifact by mixed plaque
						mLAD	Streak artifact by mixed plaque
**56/F**	100	150	417	25	384	mLAD	Partial volume with adjacent vessel
						RI[h]	Partial volume with adjacent vessel
							Low attenuation
**78/F**	80	200	604	46	754	D1	Blooming artifact by dense calcified plaque
**66/M**	100	150	399	28	1196	pRCA[i]	Blooming artifact by dense calcified plaque
**59/M**	100	100	404	23	1019	mRCA[j]	Blooming artifact by dense calcified plaque
						D2[k]	Partial volume with adjacent vessel
							Low attenuation
**75/F**	80	100	538	35	1347	mRCA	Blooming artifact by dense calcified plaque

Abbreviations: [a]Left main coronary artery, [b]Proximal left anterior descending artery, [c]Middle left anterior descending artery, [d]First obtuse marginal branch, [e]Distal left circumflex artery, [f]Proximal left circumflex artery, [g]First diagonal branch, [h]Radmus intermedius, [i]Proximal right coronary artery, [j]Middle right coronary artery, [k]Second diagonal branch.

Supplementary Table S2. False negative findings and the factors affecting these CCTA results

Age(y)/ Sex	kVp	mAs	Aorta (HU)	Noise	Calcium score	Location	Affecting factor
**66/F**	100	150	499	27	29	R-PDA[a]	Distal small branch
**67/M**	100	150	472	26	820	R-PDA	Distal small branch
**60/M**	100	150	405	36	1272	RI[b]	Distal small branch
**76/M**	100	150	505	31	365	OM1[c]	Distal small branch
**65/M**	80	200	656	24	1907	dLCx[d]	Distal small branch
**55/M**	80	150	480	28	462	mLAD[e]	Eccentric calcium
**68/M**	100	150	400	28	1021	mLAD	Eccentric calcium
						D1[f]	Distal small branch
**71/F**	100	150	395	31	414	dLCx	Distal small branch
**70/M**	100	150	501	24	2036	mLAD	Eccentric calcium
**60/M**	100	150	426	22	594	dRCA[g]	Distal small branch
						R-PDA	Distal small branch
**66/M**	100	150	399	28	1196	mLAD	Eccentric calcium
**55/M**	100	150	477	23	154	dLAD[h]	Distal small branch
						dLCx	Distal small branch
**61/M**	80	150	467	27	98	dRCA	Distal small branch

Abbreviations: [a]Right posterior descending artery, [b]Radmus intermedius, [c]First obtuse marginal branch, [d]Distal left circumflex artery, [e]Middle left anterior descending artery, [f]First diagonal branch, [g]Distal right coronary artery, [h]Distal left anterior descending artery.

Supplementary figure. The qualitative image quality assessment on a four-point scale

a) Grade 1 (poor/non-diagnostic), severe image degradation or discontinuation of vessel contour that prevented vessel lumen evaluation;

b) Grade 2 (adequate), moderate image degradation that impeded vessel lumen evaluation;

c) Grade 3 (good), minor image degradation that did not affect vessel lumen evaluation;

d) Grade 4 (excellent), no image degradation

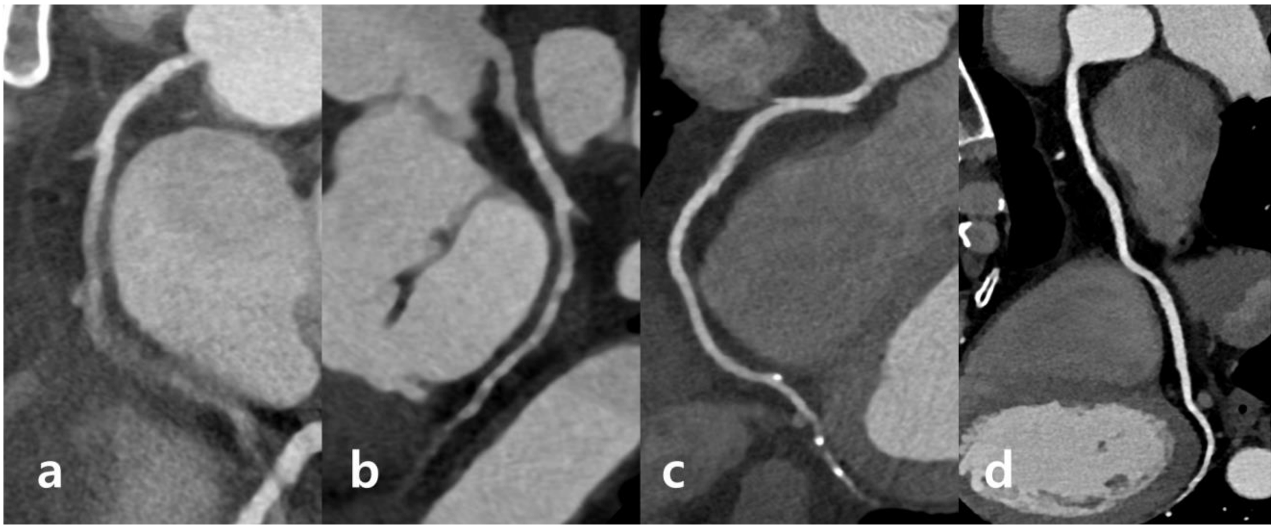

Supplement: Supplementary file 1 — Supplementary Tables and Figure [file 41598_2018_37870_MOESM1_ESM.pdf]
